# Team-based extubation protocol in cardiac surgical patients reduces ventilation time and reduces length of stay in the ICU

**DOI:** 10.1186/cc14275

**Published:** 2015-03-16

**Authors:** JM Taculod, MJ Dajac, A Del Rosario, J Gammad, S Mahaju, O Siow Eng, P Oh, R Kollengode, G Maclaren, ME Cove

**Affiliations:** 1 National University Hospital, Singapore

## Introduction

National University Hospital, Singapore, recently formed a Division of Critical Care - Respiratory Therapy. This service rapidly expanded to provide 24/7 Respiratory Therapy Services in the cardiothoracic intensive care unit (CTICU). One goal of service expansion was a reduction in duration of mechanical ventilation after cardiac surgery. We hypothesized that Introduction of a team-based extubation protocol would reduce the duration of mechanical ventilation and ultimately affect ICU length of stay.

## Methods

A multidisciplinary group created a team-based extubation protocol. The protocol was applied to all elective postoperative cardiac surgery patients. To assess the protocol's impact, data were collected in a registry 3 months before and 3 months after protocol initiation. Data collection included cardiopulmonary bypass time, McCormack airway assessment, ICU admission time, initial pH, lactate, inotropes upon arrival at the CTICU, blood gas analysis prior to extubation, time of extubation and length of stay. Patients were excluded from data analysis if they experienced events which contraindicated application of the protocol, such as significant intraoperative or postoperative complications. These events were explicitly stated in the extubation protocol. Singapore's Domain-specific review board granted waiver of patient consent to analyze and present this data.

## Results

A total of 201 patients undergoing elective open cardiac surgery were included; 99 patients before protocol implementation (pre-protocol) and 102 patients after implementation (post-protocol). There was no significant difference in mean age (60 vs. 61 *P *= 0.823), gender (79.8% vs. 79.4% *P *= 1.00), EuroSCORE (26 vs. 32 *P *= 0.576) and proportion receiving bypass surgery (72% vs. 80% *P *= 0.206) or valve surgery (21% vs. 19% *P *= 0.722) between the two groups. Median extubation time was reduced by 3.5 hours (620 minutes vs. 408 minutes *P *< 0.001). ICU length of stay was also reduced following Introduction of the pre-protocol 48 hours versus 24 hours post protocol (*P *< 0.05).

## Conclusion

A team-based extubation protocol significantly reduced the duration of mechanical ventilation and this translated to reduced ICU length of stay in patients undergoing elective open-heart surgery.

